# Developmental Perceptual Impairments: Cases When Tone-Deafness and Prosopagnosia Co-occur

**DOI:** 10.3389/fnhum.2018.00438

**Published:** 2018-10-30

**Authors:** Sébastien Paquette, Hui C. Li, Sherryse L. Corrow, Stephanie S. Buss, Jason J. S. Barton, Gottfried Schlaug

**Affiliations:** ^1^Music and Neuroimaging Laboratory, Department of Neurology, Beth Israel Deaconess Medical Center and Harvard Medical School, Boston, MA, United States; ^2^Department of Psychology, Bethel University, St. Paul, MN, United States; ^3^Human Vision and Eye Movement Laboratory, Departments of Medicine (Neurology), Ophthalmology and Visual Science, University of British Columbia, Vancouver, BC, Canada

**Keywords:** prosopagnosia, tone-deafness, amusia, developmental perceptual impairments, pitch perception, face perception

## Abstract

Studies have shown subtle gray and white matter abnormalities in subjects with several developmental disorders including prosopagnosia, tone-deafness, and dyslexia. Correlational evidence suggests that tone-deafness and dyslexia tend to co-occur, suggesting a link between these two developmental disorders. However, it is not known whether tone-deafness can also be associated with other developmental disorders such as impaired face recognition or prosopagnosia. We addressed this question by assessing face perception abilities in a group of tone-deaf individuals and matched non-tone-deaf subjects. The Cambridge (CFMT) and the Warrington (WRMT) face memory tests were used to assess face processing in the combined group of 12, out of which six tested in the tone-deaf range. Only tone-deaf participants (two out of six) scored in the impaired range on the CFMT, one of whom was also impaired on the WRMT face memory test. Furthermore, the melodic composite score of all participants on the Montreal Battery of Evaluation of Amusia significantly correlated with their face recognition score on the CFMT. Our results suggest that in some cases tone-deafness might co-occur with face recognition impairments. It is implausible that both deficits are linked to a single cognitive dysfunction that spans different perceptual systems in different modalities. They are likely associated with a common pathogenetic mechanism of early development that leads to anomalies affecting the function of different brain systems or the connection between regions.

## Introduction

Tone-deafness (Loui et al., 2011), also frequently referred to as congenital amusia (a blanket term for musical disabilities: Peretz, 2016), is a lifelong deficit in pitch discrimination and inability to sing in tune that affects approximately 1.5% (Amusia online test: Peretz and Vuvan, 2017) to 4% (Distorted Tune Test: Kalmus and Fry, 1980) of the general population. Tone-deaf individuals have impaired fine pitch discrimination, usually cannot sing in tune (Loui et al., 2011) and have difficulty recognizing melodies without the help of lyrics (Peretz, 2016). These impairments cannot be explained by hearing loss, intellectual deficiencies, or lack of exposure to music.

Tone-deafness has often been compared (Peretz, 2016) to other (seemingly) domain-specific, neurodevelopmental disorders such as developmental prosopagnosia (an impairment in recognizing face identity; estimated prevalence 2–3%: Kennerknecht et al., 2006; Bowles et al., 2009) and dyslexia (difficulty in learning to read or interpret words, letters, and symbols; estimated prevalence of 5–17.5%: Shaywitz, 1998) due to their apparent specificity and enduring nature. Other points of comparison include the fact that structural neuroimaging studies have described gray and white matter abnormalities in the inferior frontal gyrus and the arcuate fasciculus (a white matter bundle connecting temporal and frontal brain regions) of subjects with tone-deafness (right hemisphere: Hyde et al., 2006, 2007; Loui et al., 2009) and congenital dyslexia (left hemisphere: Brown et al., 2001; Silani et al., 2005). Gray and white matter abnormalities have also been observed for developmental prosopagnosia (Silani et al., 2005; Garrido et al., 2009; Song et al., 2015), but interestingly, these abnormalities are not only seen within domain-specific brain regions (e.g., areas activated by reading tasks), but also occur in other distributed brain regions [i.e., some clusters were detected by Brown et al. (2001) in the left temporo-parietal-occipital region, in the left inferior and middle temporal gyri, and inferior and superior frontal gyri].

One explanation for these partly nonoverlapping structural and functional abnormalities in both tone-deafness and prosopagnosia may be a common underlying pathology that can affect different brain regions. A possible mechanism could be an early neuronal migration disorder or problem with axonal outgrowth and/or axon guidance issues during a sensitive period in early development. In particular cases, where only one disorder is observed, a specific network vulnerability might be linked to the timing of circuit assembly in particular cortical regions (Rakic, 2002). However, such a mechanism may also produce subtle malformations in multiple brain networks, and this more widespread neural disorder may give rise to multiple functional impairments within the same individuals. Supporting this hypothesis, previously reported correlational evidence suggests an association between musical abilities and language skills (Forgeard et al., 2008; Loui et al., 2011) as well as between musical abilities and spatial processing capabilities (Douglas and Bilkey, 2007; Williamson et al., 2011). To our knowledge, no such link has yet been described between face identification and pitch discrimination impairments. Only reports of preserved emotion perception from faces and music in tone-deaf were found (Gosselin et al., 2015; Zhishuai et al., 2017).

Given the relatively low prevalence of these deficits (tone-deafness, prosopagnosia) and assuming their independence, their joint prevalence would correspond to less than 1% of the general population, when using their respective low (Bowles et al., 2009; Peretz and Vuvan, 2017) or high (Kalmus and Fry, 1980; Bowles et al., 2009) prevalence estimate.

The objective of the present study was to investigate whether congenital tone-deafness and developmental prosopagnosia co-occur, even if in some cases the phenotypical expression is subtle and the subject might not be aware of the presence of one or more disorders. To do this, we evaluated a group of tone-deaf individuals to determine if many also had a deficit in face recognition and to evaluate if this proportion is unexpected given the null hypothesis that the proportion is truly around 3% (prosopagnosia’s liberal prevalence estimate). As a second goal, we tested the hypothesis that pitch processing abilities would correlate with face recognition abilities, to further evaluate the links between these two deficits.

Previously validated tests were used to evaluate face and music perception. The Cambridge Face Memory Test (CFMT), a validated (Duchaine and Nakayama, 2006) test of short-term face familiarity, was used to quantify face recognition abilities. Tone-deaf individuals were identified and melodic perception was evaluated with the Montreal Battery for the Evaluation of Amusia (MBEA: Peretz et al., 2003). The Warrington Recognition (face and word) Memory Tests (WRMT: Warrington, 1984) were also used as additional evaluations to account for task-specific effects.

## Materials and Methods

### Participants

Participants responded to ads looking for subjects having problems singing in tune or control subjects that did not have any subjective problems with singing. Six of the subjects were not considered tone-deaf. They were matched (on gender, age, and musical background/training) to the group of subjects that were considered to be tone-deaf according to the *contour* test of the MBEA. These tone-deaf subjects were either already tested in our laboratory (and asked to come back for additional testing) or were newly recruited subjects with subjective problems singing in tune. Our criterion for tone-deafness (the MBEA contour test), previously used by Loui et al. (2009), was selected because this specific sub-test of the MBEA has shown repeatedly high correlation with our participants’ pitch perception abilities (e.g., Schlaug et al., 2015). One participant that was newly recruited for this particular study was excluded due to very poor performance across all administered tests.

The final sample consisted of six subjects that fulfilled criteria for tone-deafness and six non-tone-deaf control subjects. Age, musical training background, education level, and a correlate of overall IQ (Paulson and Lin, 1970) for all subjects are presented in Table 1: no significant differences between the tone-deaf and the control subjects were observed on these measures (Wilcoxon, all *p* > 0.05). All participants had normal audiometry thresholds for 1000, 2000, and 4000 Hz tones and reported normal or corrected vision. Nine out of 12 were native English speakers (5/6 of the tone-deaf). All volunteers gave written, informed consent, and all experiments were performed in accordance with relevant guidelines and regulations. The Institutional Review Board of Beth Israel Deaconess Medical Center, Boston, MA, United States approved this study.

**Table 1:** Group average demographic characteristics and average scores on the different behavioral assessments: MBEA, CMFT, and WRMT sub-tests (SD).

		Tone-Deaf	Controls	Wilcoxon (*p*-value)
*N*		6 (3F)	6 (3F)	–
Age		25.0 (2.1)	25.2 (4.0)	*N*s
Musical training (years)		1.7 (2.6)	2.6 (2.2)	*N*s
Education (years)		17.0 (1.1)	15.3 (1.6)	*N*s
IQ (Shipley converted to WAIS)		116.8 (4.4)	116.3 (5.0)	*N*s
**Behavioral assessments:**				
MBEA	Scale (/30)	23.2 (1.9) [3.4]	26.5 (2.8) [5.0]	*N*s [<0.05]
	Contour (/30)	19.7 (1.8) [0.9]	26.0 (2.4) [4.9]	<0.05 [<0.05]
	Interval (/30)	20.5 (3.7) [1.1]	23.7 (2.3) [3.4]	<0.05 [<0.05]
	**Melodic Composite (%)**	**70.4 (6.5)** **[1.2]**	**84.6 (4.5) [2.2]**	<0.05 [<0.05]
	Rhythm (/30)	24.5 (3.1) [3.7]	25.7 (2.3) [4.0]	*N*s [*N*s]
	^C^Below cut-off (*n*)	6	0	–
CFMT	Introduction (/18)	17.3 (1.6)	18.0 (0.0)	*N*s
	No-Noise (/30)	18.8 (7.6)	22.5 (4.2)	*N*s
	With-Noise (/24)	14.7 (5.6)	17.3 (3.1)	*N*s
	**CFMT Global (%)**	**70.6 (19.0)**	**80.3 (8.8)**	*N*s
	^C^Below cut-off (n)	2	0	–
WRMT	Words (%)	97.0 (3.0)	97.0 (4.7)	*N*s
	Faces (%)	84.3 (9.1)	86.3 (7.4)	*N*s


*^C^Cut-off scores are presented in the method section. D-prime values and comparisons are presented in brackets [] as a bias-free performance measure of the MBEA (see Henry and McAuley, 2013). Bold scores are the summation of the scores above them.*

All assessments were made in a quiet room and closed-back headphones (Sennheiser HD201) were provided for the musical assessments.

### Standardized Music Perception Assessments

The MBEA (Peretz et al., 2003) contains different tests for the assessment of separate musical components (e.g., Scale, Contour, Interval, Rhythm). All tests use the same pool of 30 novel Digitally (MIDI) produced musical phrases that were composed according to the rules of the western tonal system (mean duration 5.1 s). The short melodies are presented in pairs (30) with 2-s inter-melodies intervals and 5-s inter-trial interval; participants had to judge whether the melodies were the same or different. For half (15) of the stimuli pairs, slight manipulations have been introduced to affect pitch (scale, contour, interval) or rhythm. All tests were preceded by examples and were presented in a single session (see Peretz et al., 2003 for a more detailed description).

The scores of all tone-deaf participants were two SDs below the mean (<22) of the published (Peretz et al., 2003) normative criterion for the *contour* test of the MBEA [criterion previously used by Loui et al. (2009) for the same purpose]. In contrast, none of the control group met this criterion for tone-deafness. The two groups were significantly different on the Interval test of the same battery, and also on the melodic composite score (Scale + Contour + Interval). Crucially, all pitch base test scores were significantly different between the two groups when using scores converted to *d*-prime (see Table 1).

### Standardized Face Perception Assessments

To assess participants’ face processing abilities, the CFMT (Duchaine and Nakayama, 2006) in its upright-face version was used. The test has a practice trial followed by three test phases. The practice trial was to familiarize participants with the procedure of the first phase and consisted of cartoon faces, each presented from three different profile views after which the participant was asked to select the cartoon image they had seen on the previous screen.

In phase one, target faces were presented in the same fashion as the practice trial. Participants were to memorize faces (a left 1/3 profile, a frontal view, and a right 1/3 profile). Immediately after the learning of a face, a test trial (one memorized face and two distractors) was presented. Participants were instructed to press the key corresponding to the number below the target face (1, 2, or 3). The procedure was repeated for a total of six memorized faces.

In the second phase (no-noise), participants were presented with a single screen that had a frontal view of all six target faces they had memorized in phase one. They were given 20 s to review these faces. Following the review, participants were presented with 30 forced-choice test items (six target faces × five presentations) in a fixed, random order. Each test item contained three faces, one of which was a target face, and the participant was asked to choose the face they had memorized in phase one.

In the last phase, all faces were masked with visual noise. Participants were again presented with a review image for 20 s, then the same task was repeated but test items were presented with Gaussian noise (six target faces × four presentations).

To be considered to have face perception impairments, participants need to be two SDs below the Global score [<42.1 (58.5%)] of the CFMT (SD calculated on a group of 50 neurologically intact college-age individuals, mean age of 20.2 (see Duchaine and Nakayama, 2006 for a more detailed description).

### Complementary Face and Word Processing Tasks

Sub-components (i.e., the face and word tests) of the WRMT (Warrington, 1984) were used to assess face and word recognition in our participants. Even though the Warrington face memory test has been criticized as a measure for assessing prosopagnosia because results might be affected by the presence of non-face cues (Duchaine and Nakayama, 2006), it was used for two purposes: (1) To corroborate face-processing abilities as measured by the CFMT and (2) to control for task-specific effects or possible general memory deficits (via the word memory component) as it is proposed to do for prosopagnosia (Barton and Corrow, 2016).

Both tests were performed in our laboratory using lab computers to access the Human Vision and Eye-Movement (HVEM) laboratory website (hvemlab.org). For the face memory test, participants were presented with black and white photos of 50 unfamiliar men at a rate of one item every 3 s, and they were asked to indicate with a key press whether they found the face pleasant or unpleasant (encoding phase). Immediately following the presentation of the target photos, participants completed a two alternative forced-choice task in which they had to choose which of two faces they had previously memorized in the encoding phase. The same procedure was used for the word test. In all cases, the word test was administered before the face test. A more detailed description of both tests can be found in the WRMT manual (Warrington, 1984).

Because our participants were aged between 22 and 33, the scores were compared to the normative scores for subjects aged 18–39 provided in the WRMT manual, where scores of 38 or above are classified as normal. Additionally, calculating a discrepancy score (word minus faces) between preserved word and impaired face memory on the Warrington test can provide insight into the participant degree of face-specific perception impairment. A discrepancy score (indicating a selective deficit) is significant if it was present in less than 5% of the standardization sample (Warrington, 1984). More precisely, for faces to be significantly lower than words a difference of 10 or more is required.

## Results

### Co-occurrence of Two Disorders

Using the CFMT, we found that two (33.3%; both native English speakers) of the tone-deaf subjects, met the criterion for face recognition impairment, namely a score less than 42 (they, respectively, scored 32 and 39), whereas none of the non-tone-deaf subjects met this criterion (see Table 1).

**FIGURE 1:**
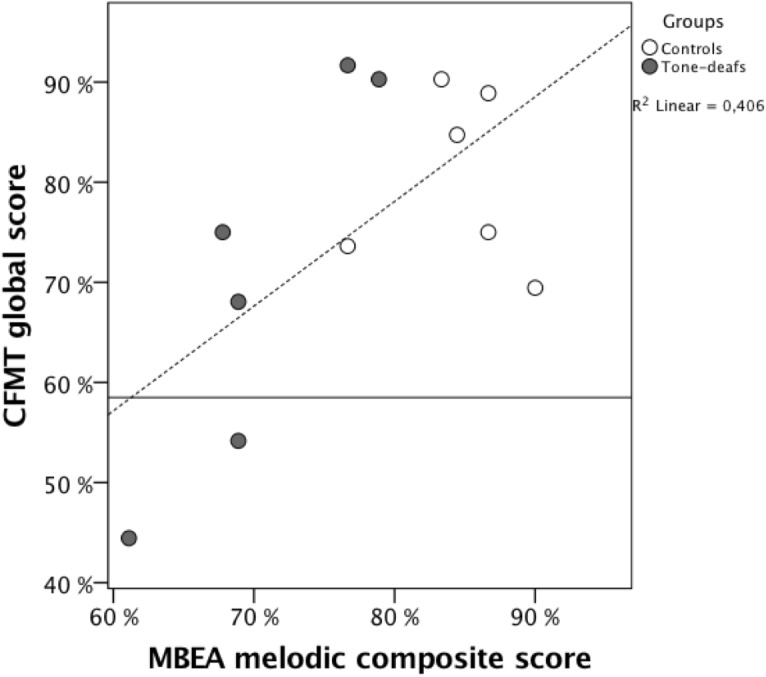
Representation of the participants CFMT global scores (*Y*) and MBEA melodic composite score (*x*) presented by groups: Tone-deaf subjects (black) and control subjects (white). The horizontal line represents the CMFT cut-off presented in text.

Using a one-sample binomial test, we were able to evaluate that the observed proportion of prosopagnosic (2/6) is significantly unexpected (*p* = 0.01) given the null hypothesis that prosopagnosia’s prevalence is truly 3% (0.18/6), the most liberal estimated prevalence published. Expanding on this link between the two measured deficits, we also found that the CFMT global score significantly correlated with the MBEA melodic composite score across all 12 individuals: *r*(12) = 0.64, *p* = 0.03 (Figure 1).

On the WRMT, all participants scored 44 or above on the word portion of the memory test. One of the two participants who scored below the cut-off on the CFMT also scored below the cut-off on the Warrington face memory tests (scored: 34; cut-off: 38). This participant also exhibited a discrepancy score of 12 (Word minus Faces), which is greater than 10 (i.e., bottom 5th percentile) indicating a selective deficit and meeting the objective component of diagnostic criteria for developmental prosopagnosia (Barton and Corrow, 2016).

Generally, participant performance on the WRMT face scores correlated with their CFMT global score *r*(12) = 0.58, *p* < 0.05, suggesting that patterns of performance on the CFMT are not specific to that test, but reflect face recognition ability in general. However, the WRMT face scores did not correlate with any MBEA sub-tests (all *p* > 0.05). This lack of correlation and the fact that we only found only one impaired score on the Warrington Face Memory Test may reflect the presence of non-face cues in this test (Duchaine and Nakayama, 2006). It is consistent with previous evidence that those with prosopagnosia can often score well on the face component of the WRMT (Duchaine, 2000; Nunn et al., 2001).

Of note, performance on the word component of the Warrington tests did not correlate with any of the face or music tests (all *p* > 0.05). Indicating that the impairments measured in the music and face perception tasks were not the result of a generalized memory deficit.

## Discussion

The present findings suggest that congenital tone-deafness might co-occur with face recognition impairments as it may co-occur with dyslexia (Forgeard et al., 2008; Loui et al., 2011) and with spatial perception deficits (Douglas and Bilkey, 2007; Williamson et al., 2011). The statistically significant difference between the observed proportion of prosopagnosia in the tone-deaf population combined with its known prevalence in the general population, and with the correlational findings, support the idea that phenotypical expressions of both disorders can co-exist. While tone-deaf subjects were identified only using the contour test of the MBEA (with the Montreal norms; Peretz et al., 2003), their MBEA melodic composite scores (Scale + Contour + Interval) were also significantly lower than their controls, highlighting their musical perception deficit. Even if tone-deafness and/or prosopagnosia were to be evaluated with other tests or if a different threshold (cut-off) was used for the MBEA (Vuvan et al., 2017), the observed correlation makes a strong point that the phenotypical expressions of both underlying abilities can be linked.

It is, however, unlikely that both deficits are linked to a single overarching cognitive dysfunction, as all participants performed well on the word recognition tests (and these results did not correlate with any other task) and all had an IQ above 110 (Paulson and Lin, 1970). This finding mirrors the observed association between musical and language abilities, suggesting a common developmental mechanism linking multiple sensory processing and recognition disorders.

While the possibility that a common neural region may be responsible for these multiple disorders remains, one current hypothesis is that the pitch perception deficit observed in the tone-deaf population may be part of a more encompassing condition that affects brain microstructural measures of white and gray matter. For example, several genes (e.g., FOXP2: Deriziotis and Fisher, 2013; ROBO1: Galaburda et al., 2006) are involved in cortical development (neuronal migration, neurite outgrowth, synaptic plasticity, and axon growth/guidance) and could be responsible for subtle cortical malformations and dysfunctions observed in these perceptual disorders (Silani et al., 2005; Hyde et al., 2006; Carter et al., 2009; Garrido et al., 2009; Loui et al., 2009; Song et al., 2015). Indeed, genetic defects or faulty expression of genes can lead to the formation of abnormal cortico-cortical and cortico-thalamic circuits that can affect different perceptual networks, and may ultimately give rise to distinct yet comorbid perceptual disorders (see Galaburda et al., 2006 for a review on the topic for dyslexia). If this is the case, perceptual impairments (i.e., tone-deafness, prosopagnosia, and dyslexia) could be observed on their own or could co-occur depending on when during cortical development a specific dysfunction occurred, this depending on the timing of circuit assembly in particular cortical regions (Rakic, 2002). The timing of the dysfunction could also possibly account for the variability in impairment/malformation observed in these perceptual disorders.

## Conclusion

Our findings suggest that tone-deafness might co-occur with other perceptual developmental disorders. We propose that this could result from common underlying neural mechanisms. Future studies with larger sample sizes, however, are required to validate the present findings. Nonetheless, these findings highlight the importance of investigating possible genetic causes or early developmental injuries that could underlie prosopagnosia and tone-deafness in order to better understand and potentially ameliorate these perceptual deficits.

## Author Contributions

GS, SC, SB, and JB developed the project. HL collected the data. SP analyzed the data, prepared the figures, and manuscript. All authors were involved in writing, editing, and reviewing the manuscript.

## Conflict of Interest Statement

The authors declare that the research was conducted in the absence of any commercial or financial relationships that could be construed as a potential conflict of interest.
